# Effect of tourniquet application on cement penetration in primary total knee arthroplasty: a meta-analysis

**DOI:** 10.1186/s42836-021-00083-7

**Published:** 2021-08-04

**Authors:** Shuxin Yao, Weijie Zhang, Jianbing Ma, Jianpeng Wang

**Affiliations:** Department of Orthopedics Honghui Hospital, Xi’An Jiaotong University , No.555 East Youyi Road, Shanxi Xi’an, China

**Keywords:** Total knee arthroplasty, Tourniquet, Cement, Meta-analysis

## Abstract

**Background:**

Tourniquet application is expected to improve surgery exposure and cementation process in total knee arthroplasty (TKA) but its effectiveness remains controversial and needs to be further explored. The aim of this meta-analysis was to assess the effect of tourniquet in primary TKA. The hypothesis is that the tourniquet application affects the cement penetration in TKA.

**Methods:**

A search was conducted in PubMed, Embase, and the Cochrane Library for the potentially eligible articles. Two independent researchers reviewed the articles retrieved against the pre-designed inclusion and exclusion criteria. In primary TKA, cement penetration was assessed, and the data between the tourniquet-assisted and non-tourniquet-assisted TKAs were compared. Statistical significance was set at *P* < 0.05.

**Results:**

A total of 4 randomized controlled trials and 3 non-randomized controlled trials (involving 675 patients) were included. There was no significant difference between the tourniquet-assisted and non-tourniquet-assisted TKAs in terms of cement penetration (*P* > 0.05). There were no significant differences in the total surgical time, blood loss, blood transfusion, the Knee Society Score, and the visual analogue scale (VAS) between the two kinds of procedures (*P* > 0.05).

**Conclusions:**

Tourniquet application may not affect cement penetration in primary TKA and may not help reduce blood loss, ease knee pain or improve the knee function. A surgeon may choose to use a tourniquet or not according to his or her own preference.

**Level of Evidence:**

Level Ib, meta-analysis.

## Introduction

Total knee arthroplasty (TKA) represents one of the most common and successful treatment alternatives for end-stage knee osteoarthritis [1]. However, TKA may fail due to a variety of reasons, including knee joint instability, infection, persistent pain, aseptic implant loosening, *etc*. [2–5]. Aseptic loosening remains the leading cause of early- and late-stage revisions. Currently, whether tourniquet use in TKA is associated with a risk of aseptic loosening remains controversial.

Aseptic loosening may be associated with individual differences, surgical techniques, and the type of implants used [4, 6]. The strength of the cement-bone interface is also important for the TKA survivorship and related revision [7–11]. Multiple *in vitro* and *in vivo* studies have shown that intraoperative bleeding and high intramedullary pressure during cement penetration might compromise the integrative and shear strength of the bone-cement interface [12, 13]. Pfitzner et al. [5] suggested a tourniquet be used in TKA because it provides a bloodless cement-bone interface, facilitates penetration of cement, improves the quality of cementation and the mechanical interlock with the implant [11]. However, some controversial studies argued that using a tourniquet may not improve cement penetration or fixation but may lead to increased blood loss, more venous thromboembolic issues, and lower functional scores of the knee in the early postoperative period [5, 14–22]. Currently, there is no convincing evidence on the effect of tourniquet on cement penetration, implant loosening, or implant survivorship.

The aim of this meta-analysis was to assess the effect of tourniquet on TKA. The hypothesis was that the tourniquet application affects cement penetration in TKA.

## Materials and methods

### Search Strategy

A meta-analysis was conducted according to the Preferred Reporting Items for Systematic Reviews and Meta-Analyses (PRISMA statement) guidelines [23]. We systematically searched the electronic databases, including PubMed, Embase, and the Cochrane Library in November 2020. The relevant English-language studies were identified. The search strategy included use of the the following terms: “Arthroplasty, Replacement, Knee”, “Tourniquet”, “Cement”, Boolean operators (AND, OR), and various combinations.

### Inclusion and Exclusion Criteria

The studies were selected against the following inclusion criteria: (1) any study on tourniquet-assisted *versus* non-tourniquet-assisted TKAs; (2) primary TKAs; (3) any report on cement penetration; and (4) research articles published in English. The exclusion criteria included: (1) review articles, case reports, letters, and comments; (2) any study on tourniquet or non-tourniquet alone; (3) cementless TKAs and (4) any report involving no comparison of results.

### Selection Criteria

The titles and abstracts of the selected articles were read, and the full text was further reviewed by two independent reviewers. A disagreement was resolved by discussion among all investigators until a final consensus was reached.

### Extraction of Data

All data of the relevant results were recorded. The data of participants included the numbers of knees and patients, demographics (age, gender, body mass index, side, *etc*.). The primary outcome measure was the cement penetration. Other relevant data, including surgical time, blood loss, blood transfusion, the Knee Society Score (KSS), and the visual analogue scale (VAS), were also extracted.

### Assessment of Quality

Two independent reviewers assessed the quality of the randomized controlled trials (RCTs) using the modified Jadad scale (7-points) on the basis of the Cochrane Handbook for Systematic Reviews of Interventions [24]. The studies that scored greater than 4 points were considered to be of high quality. The quality of the non-randomized studies was assessed on the Newcastle-Ottawa Quality Assessment Scale, which consists of 3 parts, *i*.*e*., selection (0–4 points), comparability (0–2 points), and outcome assessment (0–3 points) [25]. The studies that were awarded over 6 points were deemed of high quality.

### Statistical Analysis

Heterogeneity was determined by estimating the proportion of between-study inconsistencies by examining actual differences between studies identified in the data extraction tables. Heterogeneity was quantified using *P* and *I*². A fixed-effects model (*P* > 0.1 and *I*² < 50 %) or random-effects model (*P* ≤ 0.1 and *I²* ≥ 50 %) was used to pool the data. The study-specific odds ratio (OR) with 95 % confidence interval (CI) was employed to determine the value of dichotomous data. The continuous data were summarized as mean difference (MD) with 95 % CI using the Mantel-Haenszel method [26]. We used forest plots to graphically present the results of individual studies and the respective pooled estimate of effect size. Statistical significance was set at a *P* < 0.05. Publication bias was assessed using a funnel plot of the outcome measurement recorded in the largest number of clinical trials. Review Manager (RevMan, version 5.4) for Windows 10 and the Cochrane collaboration was utilized to perform all the statistical analyses.

## Results

### Search Results

**Fig. 1:**
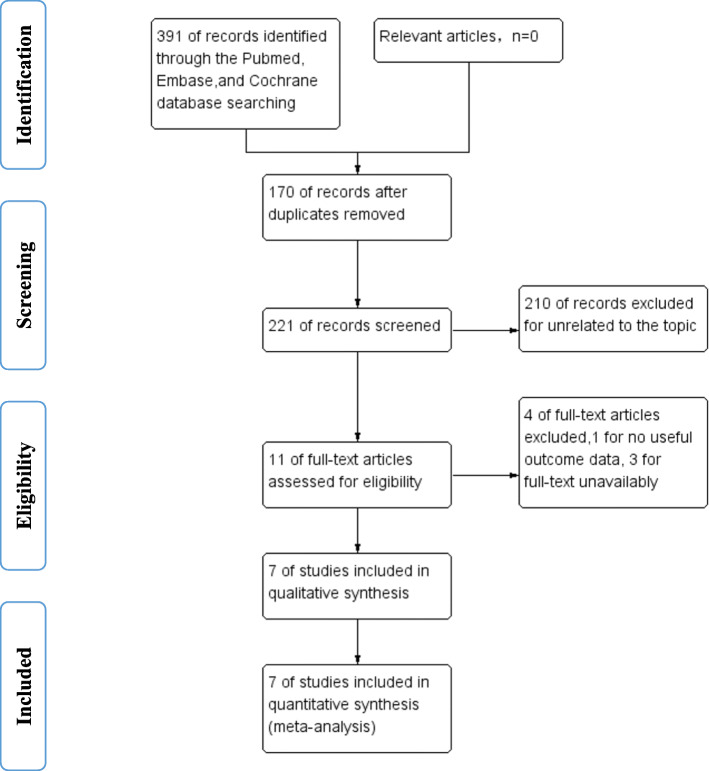
PRISMA flow diagram describing the selection process for the relevant clinical trials included in this meta-analysis

A total of 391 studies were identified. Upon reviewing of the titles, abstracts, and full articles, the unrelated articles were excluded. A total of 7 studies (4 RCTs and 3 non-RCTs) involving 675 knees were eligible and included for the final meta-analysis (Fig. 1) [5, 14, 15, 17–20]. In one study, gender was omitted; in the other 6 studies, there were 258 male and 368 female patients, with their mean ages ranging from 65 years to 71 years. Body mass index (BMI) (range, 26 to 32) was reported in 5 studies. The participants' demographics are shown in Table 1.

**Table 1:** Characteristics of 7 studies

Study	Groups	Patients (*n*)	M/F	Mean age (y)	BMI	Knee (right/left)	Cement (g)	Cement manufacturers	Tourniquet pressure (mmHg)
Pfitzner 2014 [5]	Tourniquet	45	21/24	69.3 (47–85)	27.8 (18.5–38.1)	NA	40	Palacos R^®^; Heraeus	350
	Non-tourniquet	45	11/34	70.5 (50–90)	26 (18.5–33.9)	NA			
Vertullo 2017 [14]	Tourniquet	20	10/10	67.85 ± 6.91	30.43 ± 5.07	10/10	80	Palacos R + G; Zimmer	300
	Non-tourniquet	20	11/9	65.65 ± 8.54	31 ± 5.31	13/7			
Ozkunt 2018 [15]	Tourniquet	24	NA	65.05 (52–81)	NA	NA	NA	OrCem 3; European Medical	NA
	Non-tourniquet	25	NA	65.05 (52–81)	NA	NA			
Jawhar 2018 [17]	Tourniquet	43	16/27	70 ± 6.8	31.9 ± 5.7	18/25	40	SmartSet; DePuySynthes	360
	Non-tourniquet	43	16/27	71 ± 6.8	31.9 ± 5.7	26/17			
Touzopoulos 2019 [18]	Tourniquet	50	42/8	70.73 ± 6.56	31.04 ± 5.43	NA	20	Palacos R + G^®^; Heraeus	350
	Non-tourniquet	50	42/8	69.92 ± 6.89	31.12 ± 3.95	NA			
Herndon 2019 [19]	Tourniquet	70	28/42	67 ± 9.2	NA	NA	80	Simplex; Stryker	250
	Non-tourniquet	70	26/44	67.5 ± 8.3	NA	NA			
Dincel 2020 [20]	Tourniquet	74	15/59	65.34 ± 7.94	32.83 ± 5.80	40/34	NA	Hi-Fatigue; Zimmer Biomet	150 + systolic pressure
	Non-tourniquet	96	20/76	66.12 ± 8.78	32.72 ± 5.73	55/41			

*NA* not available; *BMI* body mass index; *y* year; *g* gram

### Risk of Bias Assessment

**Fig. 2:**
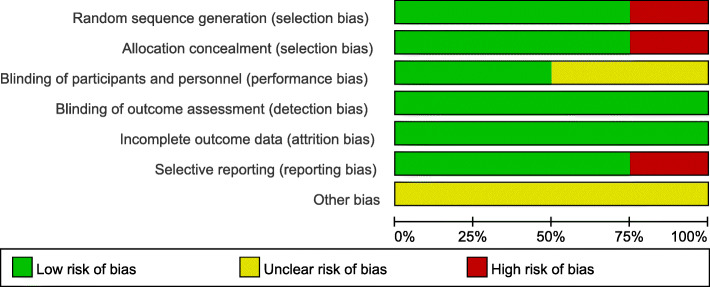
Risk of bias graph for Randomized Controlled Trials (RCTs)

**Fig. 3:**
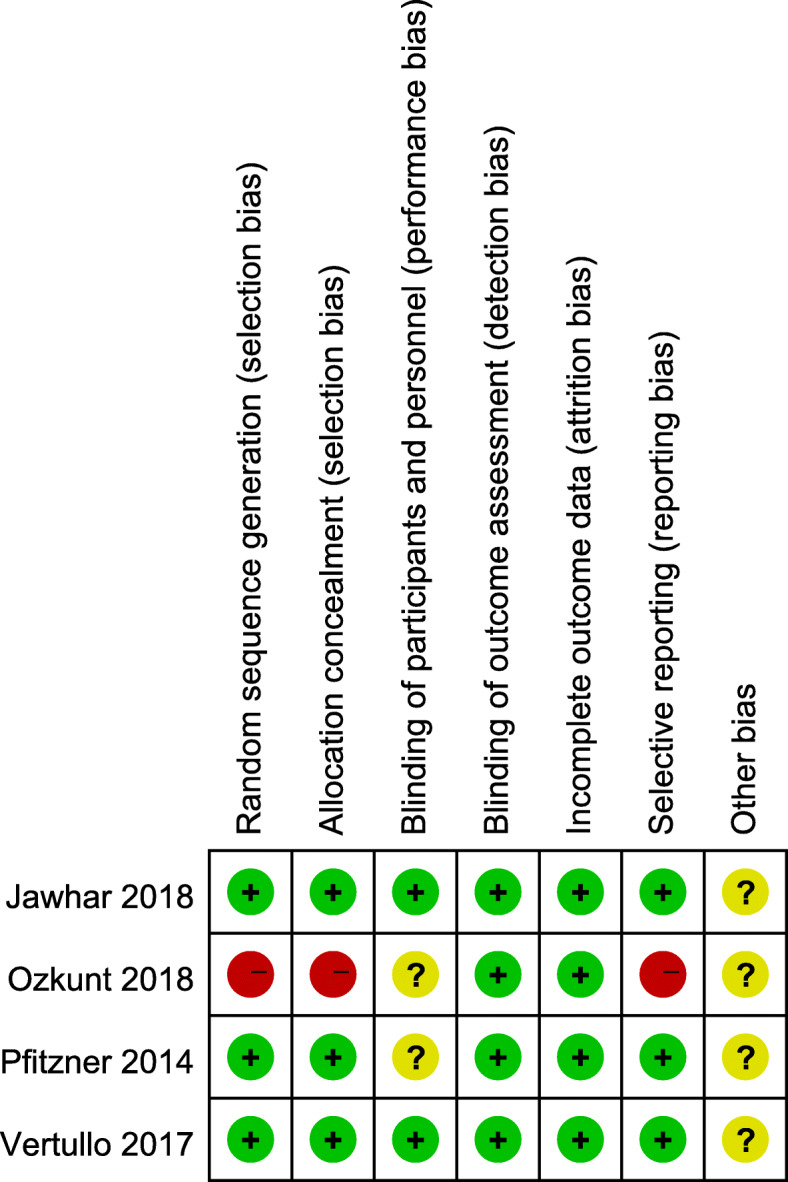
Risk of bias summary for Randomized Controlled Trials (RCTs)

All the RCTs provided clear inclusion and exclusion criteria, which suggested that methodologically randomization was used. Randomization algorithm was generated by computer in 2 studies, sealed envelopes were used in 1 study, and the order of admission to the hospital was used in 1 study. The surgeons were blinded in 2 studies. Clearly selective outcomes were reported in 6 studies. The outcome assessments were blinded in all RCTs. The results are summarized in Figs. 2 and 3. The Newcastle-Ottawa Scale, including selection, comparability, and exposure, was used to assess the retrospective studies. All the 3 studies showed good patient selection, unrelated variable control, and result reporting. Since all tourniquet and non-tourniquet groups were controlled in the hospital, they only got 3 stars (points) at the first part. The results are listed in Table 2.

**Table 2:** Quality assessment of 3 non-randomized controlled trials

Studies	Newcastle-Ottawa Scale			Quality
	Selection	Comparability	Exposure	
Touzopoulos 2019 [18]	***	**	***	High
Herndon 2019 [19]	***	**	***	High
Dincel 2020 [20]	***	**	***	High

**, scored 2 points; ***, scored three points

### Meta-analysis

**Fig. 4:**
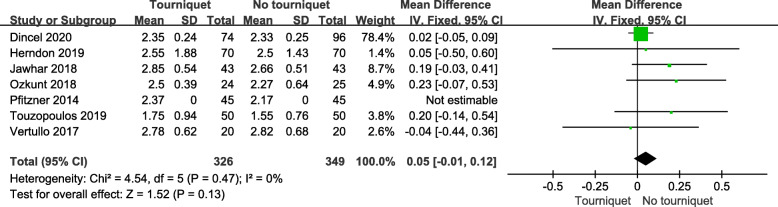
Forest plot of pooled cement penetration among included studies

Cement penetration in the tourniquet and non-tourniquet groups was compared in 7 studies, and the data were expressed as mean ± standard deviation in 6 studies. To make it comparable, we calculated the mean values of the depth of cement penetration (range, 1.55–2.85 mm). There was no significant statistical difference between the tourniquet and non-tourniquet groups (*P* = 0.13; MD = 0.05; 95 % CI, -0.01 to 0.12; Fig. 4).

**Fig. 5:**
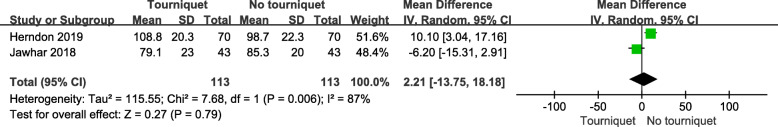
Forest plot of pooled surgical time between included studies

Surgical time was recorded in 2 studies, but the difference between the 2 studies was evident due to the different surgeons and surgical skills. However, the pooled data were still comparable, and showed no statistically significant difference between the tourniquet and non-tourniquet groups (*P* = 0.79; MD = 2.21; 95 % CI, -13.75 to 18.18) (Fig. 5).

**Fig. 6:**
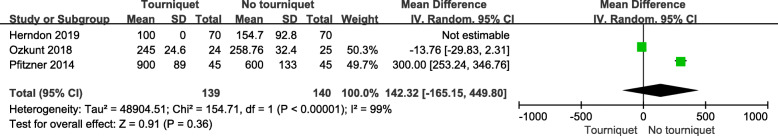
Forest plot of pooled Blood loss among included studies

**Fig. 7:**
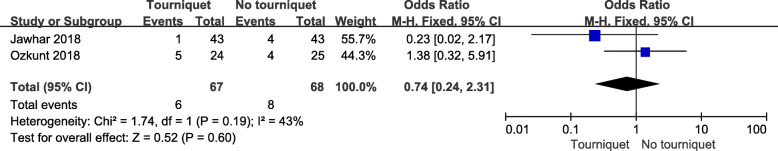
Forest plot of pooled blood transfusion between included studies

Postoperative drainage or estimated total blood loss was reported in 3 studies, and transfusion was recorded in 2 studies. There were no statistically significant differences between the tourniquet and non-tourniquet groups in terms of blood loss or transfusion (*P* = 0.36; MD = -142.32; 95 % CI, -165.15 to 449.80; and OR = 0.74; 95 % CI, 0.24 to 2.31; *P* = 0.60) (Figs. 6 and 7).

**Fig. 8:**
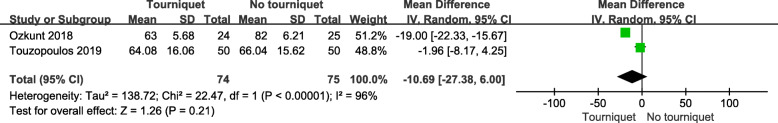
Forest plot of pooled knee society score (KSS) between included studies

The KSS was used to assess the range of motion and knee function in 2 studies. Although the scores of the non-tourniquet groups had a cumulative increase of 10.69 points, the pooled data showed no statistically significant difference between the tourniquet and non-tourniquet groups (*P* = 0.21; MD = -10.69; 95 % CI, -27.38 to 6.00) (Fig. 8).

**Fig. 9:**
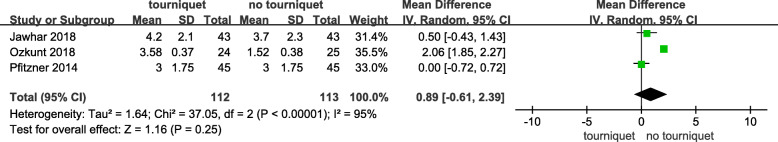
Forest plot of pooled visual analogue scale (VAS) among included studies

The VAS was used to evaluate postoperative knee pain in 3 studies (225 patients). The VAS of the non-tourniquet group was 0.89 points, which was higher than that of the tourniquet group but the difference was not statistically significant (*P* = 0.25; MD = 0.89; 95 % CI, -0.61 to 2.39) (Fig. 9).

**Fig. 10:**
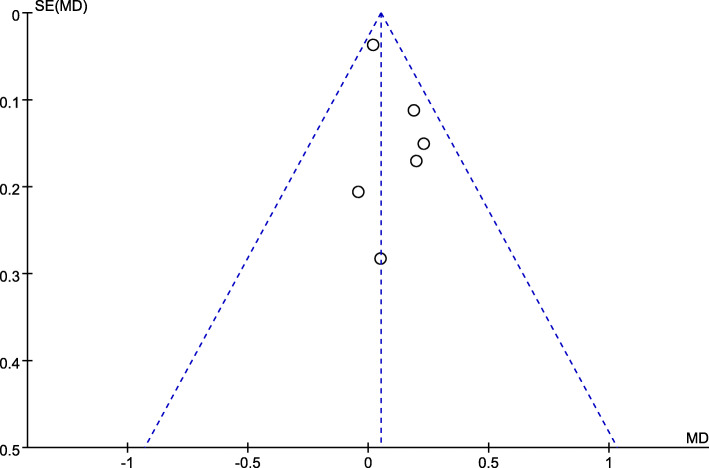
Funnel plot demonstrating a minimal publication bias of cement penetration

Publication bias was assessed by creating a funnel plot, which demonstrated the relationship between the sample size of the studies and the precision in the estimation of the treatment effect. The result showed no substantial evidence of publication bias in cement penetration (Fig. 10).

## Discussion

The most important finding of this meta-analysis was that both the tourniquet-assisted and non-tourniquet-assisted TKAs yielded very similar results in terms of cement penetration, surgical time, blood loss, transfusion, KSS, and VAS.

The implant stability is intimately associated with the depth of cement penetration [27, 28]. For cement to reach the first transverse trabeculae, 2–3mm penetration was required [8]. Walker *et al*. [28] suggested that the optimal depth of cement penetration is 3 to 4 mm for maximal cement-bone interface fixation. In our study, however, the mean cement penetration depth ranged from 1.55 mm to 2.85 mm, which might vary with operative skills of different surgeons.

So far, tourniquet use in TKA remains controversial. Touzopoulos *et al*. [18] demonstrated the average penetration at all levels was less than 2 mm in the tourniquet-assisted TKA, whereas the penetration in the non-tourniquet-assisted TKA was 1.2 mm cumulatively. In a randomized, prospective clinical trial, Pfitzner *et al*. [5] evaluated the cement mantle of the tibial component in primary TKA and found the use of a tourniquet increased the tibial cement mantle thickness by1.2 mm. Hofmann *et al*. [29] identified a 2.69 mm overall depth of penetration in 109 patients and their mid-term follow-up revealed excellent durability. Ozkunt* et al*. [15] found an average of 2.35 mm penetration, and the use of tourniquet had no effect on cement penetration. Furthermore, some surgeons radiosterometrically compared the short-term effect on implant stability and failed to find significant difference in terms of implant stability between the two groups [16, 30, 31].

Reducing bleeding is one of the reasons for using a tourniquet in TKA. In a recent systematic review involving 25 RCTs, Moher *et al*. [32] showed the use of tourniquet significantly decreased intraoperative blood loss but might not reduce the total blood loss. Li *et al*. [33] retrospectively compared the tourniquet-assisted TKAs to the non-tourniquet-assisted ones, and found no difference in perioperative blood loss or postoperative blood transfusion. Pfitzner *et al*. [5] found that blood loss was more in tourniquet-assisted TKAs.

In the past, the application of a tourniquet in TKA was also believed to be able to shorten surgical time. In a prior meta-analysis involving 13 RCTs (859 patients), Yi *et al*. [34] demonstrated that tourniquet use could reduce the surgical time. Mutlu* et al*. [35] reported similar results in a retrospective cohort study. However, different viewpoints have been proposed in more studies. In a randomized study of 70 patients, Ejaz *et al*. [36] showed the tourniquet group and the non-tourniquet group took similar surgical time (71 ± 4.5 min vs. 70 ± 5.3 min). Herndon *et al.* [19] reported a longer surgical time in the tourniquet group (109 min) than in the non-tourniquet group (99 min). In the present study, we did not find a significant difference because only limited studies were included.

In a prospective, randomized study, Zhao *et al*. [37] showed that the KSS was significantly better in the non-tourniquet group 3 weeks after surgery but no significant difference was found after 3 months. In another prospective randomized study, Ozkunt *et al*. [15] did not observe any statistically significant differences in preoperative KSS between the long-duration tourniquet group, short-duration tourniquet group, and non-tourniquet group. However, a significantly worse postoperative KSS was found in the long-duration tourniquet group. Furthermore, in a recent comparative study, Touzopoulos *et al*. [18] found no significant difference in KSS at the final follow-up. From those limited studies, we are led to conclude that there existed no difference between tourniquet-assisted and non-tourniquet-assisted TKAs.

Olivecrona* et al*. [38] showed that long tourniquet time (over 100 min) raised the risks of complications caused by oxygen deprivation of the soft tissues, ischemia-reperfusion injury, local inflammation, muscle injuries, and knee pain. Oxygen-free radicals and inflammatory factors (neutrophils, tumor necrosis factor α, and interleukin 8) are also important contributors [39]. Excessive use of a tourniquet and increased pressure applied lead to swelling and congestion of the bone compartment and might lead to rhabdomyolysis [40]. Ejaz *et al*. [36] found that complications were less in non-tourniquet-assisted TKAs.

Jawhar *et al*. [17] performed 86 primary TKAs. They found deep vein thrombosis in one patient and did one revision surgery due to surgical site infection in the tourniquet group. In the non-tourniquet group, one patient had a delayed wound healing. There was no significant difference between the two groups in the complications.

This study has some limitations. First, the low level of evidence of the 3 non-RCTs might lead to statistical bias and involve other confounding variables. Second, the publication bias might affect the outcomes. Third, the limited studies and different standards on cement penetration rendered the data less comparable. Finally, the differences in surgical techniques, bone densities, and cement used might impact the final results. Future high-quality RCTs are warranted to illustrate the exact effect of tourniquet on TKA outcomes.

## Conclusions

Tourniquet application may not improve cement penetration in TKA and may not offer benefits for reducing blood loss, easing knee pain or improving the knee function.

## Data Availability

The datasets used or analyzed during the current study are available from the corresponding author on reasonable request.

## Abbreviations

ERAS: Enhanced recovery after surgery
TKA: Total knee arthroplasty
RCTs: Randomized controlled trials
KSS: Knee society score
VAS: Visual analogue scale
OA: Osteoarthritis
PRISMA: Preferred Reporting Items for Systematic Reviews and Meta-Analyses guidelines
NOS: Newcastle-Ottawa Quality Assessment Scale
OR: Odds Ratio
MD: Mean difference
CI: Confidence intervals
